# Arbuscular mycorrhizal fungi decouple plant growth and vanadium accumulation by reprogramming vanadium dynamics in green foxtail (*Setaria viridis*)

**DOI:** 10.3389/fpls.2025.1731846

**Published:** 2026-02-12

**Authors:** Ze Xi, Jinlong Wang, Yue Zhou, Yuexiao Dong, Shah Kinjal J., Shujuan Zhang

**Affiliations:** 1 College of Urban Construction, Nanjing Tech University, Nanjing, China; 2 Nanjing Yuqing Environmental Technology Co., Ltd, Nanjing, China

**Keywords:** AMF, cell wall, phytochelatins, translocation, vanadium

## Abstract

**Introduction:**

The urgent need for bioremediation strategies in vanadium(V)-polluted soils necessitates deeper insights into the role of arbuscular mycorrhizal fungi (AMF) in enhancing growth and V accumulation in green foxtail (*Setaria viridis*).

**Methods:**

Using *Rhizophagus irregularis* as the tested AMF strain, we created two treatments under V stress (200 mg kg^-1^): inoculation with AMF and non-inoculation, to investigate how AMF colonization influences growth and V accumulation of green foxtail.

**Results:**

We found that under V stress, AMF inoculation increased leaf width by 27%, plant height by 60%, shoot biomass by 109% and root biomass by 104%. In addition, AMF decreased V concentrations across tissues: roots by 73%, chlorophyllous shoots by 74%, achlorophyllous shoots by 88%, and panicles by 94%. The biological enrichment factor was 0.4 for inoculated plants and 1.6 for non-inoculated plants while the translocation of V from roots to panicles and leaves was decreased by AMF inoculation. Subcellular analysis revealed preferential V sequestration in cell walls, accounting for 72% (inoculated) vs. 78% (non-inoculated) of total cellular V, with leaf cell wall concentrations being 34% lower in colonized plants. Additionally, AMF raised the levels of phytochelatins in roots by 8% and non-protein thiols by 31% and glutathione in both shoots (by 37% in leaves, 4% in stems) and roots (by 121%).

**Discussion:**

More focus should be placed on AMF in the bioremediation of V-polluted soil as they decoupled plant growth and V accumulation by altering enrichment, translocation, subcellular distribution and chelation of V in green foxtail.

## 1 Introduction

Soil vanadium (V) pollution in some regions of China poses severe ecological risks, demanding innovative bioremediation strategies. Industries like steel production, battery manufacturing, and petroleum refining all make extensive use of V (Hao et al., 2015; Wolowicz et al., 2022). Anthropogenic activities spanning V mining, smelting, consumption and waste disposal continuously release substantial amounts of V into soils, leading to widespread V pollution (Yin et al., 2022). Xiao et al (Xiao et al., 2015). discovered that V concentrations in soils close to a stone coal site in Hunan ranged from 168 to 1538 mg kg^-1^, surpassing the limit of 130 mg kg^-1^ set by Canada and the background level of 82 mg kg^-1^ of China. Similarly, soil samples from the Panzhihua smelting region in China have shown V concentrations as high as 1130.10 mg kg^-1^ (Zhang et al., 2019). In some areas of China, soil V concentrations exceeded the land use screening values for Category I construction land (165 mg kg^-1^) and Category II (752 mg kg^-1^) (Hao et al., 2021). Furthermore, urban parks are also affected by soil V pollution, in addition to V mining and smelting areas (Teng et al., 2011). More significantly, plants, animals, and soil microorganisms are toxically affected by the slightly higher dose of V (133 mg kg^-1^) (Xiao et al., 2012; Gustafsson, 2019; Xia et al., 2022), which poses a risk to human health (Li et al., 2020).

To improve phytoremediation of V-polluted soils, various strategies have been developed to improve plant tolerance to V, including plant selection, microbial augmentation, and the application of exogenous additives. During plant selection, green foxtail was shown to have stronger accumulation of multi metal pollution (V, Cr, Zn and Cu) and higher potential for use as a phytoremediation plant at multi-metal polluted sites than other tested plants such as lamb’s-quarters (*Chenopodium album*), carrot (*Daucus carota*) and indian fieldcress (*Rorippa indica*) (Aihemaiti et al., 2017). Furthermore, between two mustard genotypes under V stress, the purple genotype showed higher tolerance to V than the green genotype (Imtiaz et al., 2018). For microbial augmentation, endophyte inoculation mobilized V by promoting the secretion of plant organic acids, increasing V immobilization on the root surface, strengthening plant antioxidant systems, and promoting V(V) reduction to V(IV) in the roots (Wang et al., 2023). The researchers also found evidence that soil native microorganisms such as functional genera (e.g. *Aliihoeflea, Actinotalea*) converted soluble V(V) to insoluble V(IV) and reduced the release of dissolved V from V-containing dusts (Li Y. N. et al., 2022). Regarding the application of exogenous additives, liquid digestate has been reported to increase seed germination and seedling growth of green pigweed, which is due to a combined effect of direct reduction of V(V) species and improvement of plant nutrition (Aihemaiti et al., 2019a). In addition to liquid digestate, solid olive waste, selenium nanoparticles (Albqmi et al., 2023), boron, melatonin (Nawaz et al., 2018; Shireen et al., 2021) and exogenous 3,3’-diindolylmethane (Gokul et al., 2021) have been used to alleviate V toxicity in plants. The above methods have improved plant tolerance to V to varying degrees. While physical-chemical remediation strategies are widely employed, their high operational costs, secondary pollution risks, and disruption of soil microbiota severely limit sustainable implementation (Wang X. W. et al., 2020).

The research on the function of plants in remediating V-polluted soils has been predicated on the knowledge that a plant’s underground portion is solely made up of its roots and does not form symbiotic relationships with soil microorganisms (Aihemaiti et al., 2020; Chen et al., 2021). In reality, the majority of plant roots form symbiotic structures with endophytic bacteria (Wang et al., 2018, Wang L. et al., 2020) and arbuscular mycorrhizal fungi (AMF) (Jiang et al., 2017). AMF can form arbuscular mycorrhizae with about 200,000 plant species (or about 80% of terrestrial plant species) and belong to the phylum *Glomeromycota*, class *Glomeromycetes*, and consist of 4 orders, 12 families, and 36 genera (Wang et al., 2021). Growing interest has been shown in the role of AMF in remediating soils polluted with heavy metals, such as As, Cr and Cd (Sharma et al., 2017; Ahammed et al., 2023; Fang et al., 2024). The mechanisms of AMF ranged from molecular chelation, subcellular partitioning, and translocation improvement (Dhalaria et al., 2020; Wahab et al., 2023; Zhuang et al., 2025). There are some studies reporting the role of AMF in remediating V-polluted soils (Qiu et al., 2021; Selim et al., 2021). Some of hostplants of AMF—like green foxtail, are V hyperaccumulator candidates (Aihemaiti et al., 2017; Zhang et al., 2017). Importantly arbuscular mycorrhizae were found to form in V-polluted soils and improved V tolerance of host plants (Aihemaiti et al., 2019b; Zhang et al., 2025a). Collectively, AMF show promise in V phytoremediation, yet their mechanisms governing V behaviors remain underexplored.

We aimed to investigate whether and how AMF improve plant growth and V accumulation. Our previous studies found that the stain of *Rhizophagus irregularis* enhanced plant tolerance to V stress by protecting the ultrastructure of leaf cells, improving the antioxidant system and inducing a growth dilution effect (Zhang et al., 2025a). In addition, this strain improved soil physical and chemical properties by not only increasing soil aggregation, total organic carbon concentrations and concentrations of glomalin-related soil protein, but also decreasing soil pH (Zhang et al., 2025a). Given its tolerance to high V pollution (Zhang et al., 2025b) and commercial availability (Ceballos et al., 2013), *Rhizophagus irregularis* is a robust candidate strain for the remediation of V-polluted soils. We hypothesize that AMF colonization (1) increases enrichment and root-to-shoot V translocation, (2) sequesters more V in cell walls and vacuoles, and (3) upregulates thiol-based chelators.

## 2 Materials and methods

### 2.1 Plant, fungi and substrates

Green foxtail (*Setaria viridis*) is the plant material used, and Jiangsu Xintai Seed Industry Wholesale Co., China provided the seeds. AMF can readily colonize the roots of the green foxtail which is a common mycorrhizal plant and form symbiotic relationships with it (Zhang et al., 2017). The Shanghai Institutes for Biological Sciences provided the AMF strain (*Rhizophagus irregularis*), which had a spore density of 76 spores per 100 grams and the percentage of root length colonization was 42%. Soil and quartz sand with diameters varying from 1 to 2 mm, made up the substrate. *Rhizophagus irregularis* was selected for this study due to its efficiency in reducing plant metal accumulation and enhancing biomass, as detailed in the Introduction. Its tolerance to high V pollution and commercial availability make it an ideal candidate for V-polluted soil remediation (Ceballos et al., 2013; Zhao et al., 2024; Zhang et al., 2025a, Zhang et al., 2025a). Henan Water Source Water Purification Materials Co., Ltd was the supplier of the quartz sand. After being repeatedly cleaned with tap water, the quartz sand was dried in an oven to a consistent weight. The State Experimental Station of Agro-Ecosystem in Changshu, which is situated at 31°32′55 N, 120°39′24 E, provided the experimental soil, where the concentration of soil V was not detectable. For storage, the gathered soil was ground, sieved (2 mm), and allowed to air dry naturally in the lab. Before being used, the soil and quartz sand were both autoclaved for two hours at 121 °C to sterilize them. The substrates were then made by combining the sterilized soil with quartz sand in a 4:6 ratio. To reach the intended V concentration of 200 mg kg^-^¹, V was added in the form of sodium metavanadate solution, thoroughly mixed, and then aged for 50 days prior to use. The nutrient-rich soil purchased from the Xingyue Flagship Store consisted of imported peat, imported low-salt coconut coir, perlite, nitrogen-phosphorus-potassium fertilizer (N-P_2_O_5_-K_2_O: 15-15–15 and the total nutrient ≥45%), vermiculite and chlorophyll nutrients. We added 0.2 kg of nutrient soil per pot to promote plant growth. The substrate contained 1.13 g kg^-1^ total nitrogen, 10.6 g kg^-1^ soil organic carbon and 0.35 g kg^-1^ total phosphorous at the beginning of the experiment. Plants received 50 mL deionized water per pot every 3 days.

### 2.2 Experimental design

Indoor pot culture was used for the experiment, and green polyethylene plastic pots measuring 11 cm in diameter and 13 cm in height were used. AMF inoculation (+AMF) and no AMF inoculation (-AMF) were included in the experimental design. Two treatments, either +AMF or -AMF, were established under V stress. For each treatment, there were five biological replicates. Initially, 800 g of substrates were put into each pot, and then 150 g of substrates were well combined with 50 g of inoculum, for a total of 1000 g per pot. For the non-inoculated treatment, 50 g of control inoculum without AMF was used as a substitute. To avoid pollution from other microorganisms, 0.4 g of green foxtail seeds were soaked in 75% alcohol before being planted in each pot. 50 mL of water was added to each pot every three days. The experiment was conducted in a greenhouse with conditions set to a photosynthetic photon flux density (PPFD) of 102 ± 3 μmol m^-2^ s^-1^ at canopy level, daytime temperature of 28 °C for 12 hours, and nighttime temperature of 24 °C for 12 hours. After being sown, the green foxtail seeds were cultivated for two months before being destructively harvested.

### 2.3 Sample collection and measurement

Leaf width was determined before destructive harvest. Three fully expanded, representative leaves per plant were selected, and the maximum width perpendicular to the midrib was measured to the nearest 1 mm with a digital caliper. Plant height was recorded as the vertical distance from the soil surface to the tip of the tallest culm, measured to the nearest 1 mm with a rigid metre rule.

Immediately after harvest, all tissues were rinsed thoroughly with ice-cold deionized water to remove adhering soil and surface contaminants. Each plant was dissected into roots, stems, and leaves. To eliminate extracellular V that might have precipitated on the rhizodermis, root segments were immersed in 10 mM Na_2_-EDTA (pH 8.0) for 20 min on ice, then rinsed twice with deionized water. Excess moisture was removed by blotting on lint-free paper. Roots were finely chopped and pooled, then aliquoted into 2.0 ± 0.05 g portions; stems and leaves were aliquoted into 3.0 ± 0.05 g portions. All fresh subsamples were stored at -20 °C until analysis.

Immediately after dissection, root and shoot samples were subjected to a two-step drying protocol. First, tissues were transferred to a pre-heated forced-air oven at 105 °C for 30 min to arrest enzymatic activity and prevent respiratory losses. After cooling to ambient temperature in a desiccator, samples were dried at 70 °C to constant mass (± 0.1% change over two consecutive 3-h intervals). Each tissue type was weighed separately on an analytical balance (precision 0.0001 g), and the final dry weight was recorded as the biomass of roots and shoots.

Fresh green foxtail root samples were taken as part of the evaluation process for AMF colonization in green foxtail roots. The samples were then decolored for 30 minutes in a boiling water bath containing 10% potassium hydroxide. Following five minutes of soaking in a 2 percent hydrochloric acid solution, the samples were rinsed with distilled water and stained for thirty minutes in a 90 °C water bath using a 0.01% acidic fuchsin–lactic acid–glycerol solution. The samples were examined under a microscope, and the gridline intersect method was used to determine the percentage of root mycorrhizal colonization (Giovannetti and Mosse, 1980).

To determine V concentration, dried shoot and root samples were milled to a fine powder. Exactly 0.5000 g of powder was transferred into a Teflon microwave vessel and digested with 3 mL concentrated HNO_3_ plus 9 mL concentrated HCl (aqua regia) in a fume hood. Digestion was performed in a MARS-5 microwave system (CEM, USA) with the following programme: ramp to 120 °C in 10 min, hold for 10 min at 400 W; ramp to 150 °C in 10 min, hold for 30 min at 1000 W. After cooling, the clear digests were diluted to 40 mL with 1% (v/v) HNO_3_, filtered (0.45 µm, PTFE), and analyzed for V by inductively coupled plasma mass spectrometry (ICP-MS).

Furthermore, the assessment of V migration and transformation within the soil-plant system is made possible by the introduction of the enrichment and translocation factors. The three parts of a green foxtail shoot are panicles, achlorophyllous shoots, and chlorophyllous shoots. The translocation factor and enrichment factor were computed using Equations 1–4 (Qiu et al., 2021).

(1) $${\text{Enrichment factor}}_{\text{root}\leftarrow \text{substrate }}=\frac{C_{root}}{C_{substrate}}$$

(2) $${\text{Translocation factor}}_{\text{chlorophyllous shoot}\leftarrow \text{root }}=\frac{C_{chlorophyllous\;shoot}}{C_{root}}$$

(3) $${\text{Translocation factor}}_{\text{achlorophyllous shoot}\leftarrow \text{root }}=\frac{C_{achlorophyllous\;shoot}}{C_{root}}$$

(4) $${\text{Translocation factor}}_{\text{panicle}\leftarrow \text{root }}=\frac{C_{panicle}}{C_{root}}$$

Where *C_root_*, *C_substrate_*, *C_chlorophyllous shoot_*, *C_achlorophyllous shoot_* and *C_panicle_* denote the V concentration in root, substrate, chlorophyllous shoots, achlorophyllous shoots and panicles, respectively (mg kg^-1^).

Fresh leaf (1.000 ± 0.005 g) and stem (1.000 ± 0.005 g) were processed separately. Each tissue was homogenized in 20 mL ice-cold deionized water with a pre-chilled mortar and pestle, transferred to a 50 mL centrifuge tube, and combined with 20 mL extraction buffer (0.25 mol L^-1^ sucrose, 50 mmol L^-1^ Tris-HCl, pH 7.5, 1 mmol L^-1^ dithiothreitol). Subcellular fractions were obtained at 4 °C by differential centrifugation (Howell et al., 1989):

- Cell-wall debris & intact cells 200 g min^-1^, 10 min → pellet (P1)
- Organelles (mitochondria, chloroplasts) 10–000 g min^-1^, 30 min → pellet (P2)
- Soluble fraction final supernatant (S3)

To quantify V in the subcellular fractions of green foxtail, a 10 mL aliquot of each fraction was transferred to a 100 mL borosilicate Erlenmeyer flask and pre-digested overnight in 25 mL concentrated HNO_3_ (65%, w/w). The next morning, 5 mL H_2_0_2_; (30%, w/w) was added dropwise, and the flask was heated on a hot plate (200 °C) under a short-stem glass funnel until the solution became colourless to faint yellow. Whenever the volume fell below 5 mL, 1–2 mL fresh aqua regia (HNO_3_: HCl = 1:3, v/v) was added, and heating continued until dense white fumes appeared. After cooling, 2 mL ultrapure water was added, the walls were rinsed, and the solution was gently re-heated to near-dryness to remove residual acid. The digest was cooled, quantitatively transferred to a 25 mL volumetric flask, brought to volume with 1% (v/v) HNO_3_, and filtered through 0.45 µm PTFE. Vanadium was measured by ICP-MS, with GBW-10015 (spinach) as the certified reference material; recoveries were 95–105% (Bednar, 2009).

Non-protein thiols (NPT) were quantified colorimetrically using Ellman’s reagent (DTNB). Fresh plant tissue (1.000 ± 0.005 g) was ground in 4 mL ice-cold 0.5% (w/v) sulfosalicylic acid containing a pinch of acid-washed quartz sand. The slurry was transferred to a 1.5 mL micro-centrifuge tube and centrifuged at 8–000 g min-1 for 15 min at 4 °C. A 1 mL aliquot of the supernatant was combined with 0.15 mL 10 mmol L^-^¹ DTNB and 3.05 mL 0.25 mol L^-^¹ Tris-HCl (pH 8.3) in a 10 mL glass tube. After 20 min at 25 °C, absorbance at 412 nm was read against a reagent blank on a UV/Vis spectrophotometer. NPT concentrations were calculated from a GSH standard curve (0–1.0 mmol L^-^¹, R² ≥ 0.999) and expressed as µmol g^-^¹ FW (Bhargava et al., 2005).

Using the DTNB colorimetric method, the concentration of GSH was determined by weighing 1 g of fresh plant material and adding 5 mL of 0.2 mmol L^-^¹ phosphate buffer solution (pH 7.5) and a small amount of quartz sand for grinding. Split the resultant extract in half, then reacted each half with formaldehyde for two and sixty minutes, respectively, at pH 8. After adding 5 mL of DTNB solution to 1 mL of each reaction, let them react for 5 minutes at 25 °C. Calculated the difference between the two absorbance values after measuring each sample’s absorbance at a wavelength of 412 nm. Based on this discrepancy, determined the GSH concentration using the standard curve (Castillo and Greppin, 1988).

The concentration of PCs (Equation 5) was determined using the differential method, which involves the calculation:

(5) $$C_{PCs}=\;C_{NPT}-\;C_{GSH}$$

Where *C_PCs_*, *C_NPT_* and *C_GSH_* refer to the concentrations of PCs, NPT and GSH respectively, in (mg g^-1^).

### 2.4 Statistical analysis

All data were evaluated for homogeneity of variance and normality. The statistical program SPSS (SPSS Inc.) was used to analyze the experimental data. Chicago, USA) in order to conduct the analysis. RStudio was used to create scatter plots and bar charts (RCoreTeam, 2024).

## 3 Results

### 3.1 AMF colonization

In our pot experiment, plants were subjected to treatments either with AMF inoculation (+AMF) or without AMF inoculation (-AMF). Colonization was observed in +AMF plant root while no colonization was observed in -AMF plant roots. The percentage of root length colonization of AMF of inoculated plants was 39% (± 2%).

### 3.2 V concentrations of shoot and root

**Figure 1:**
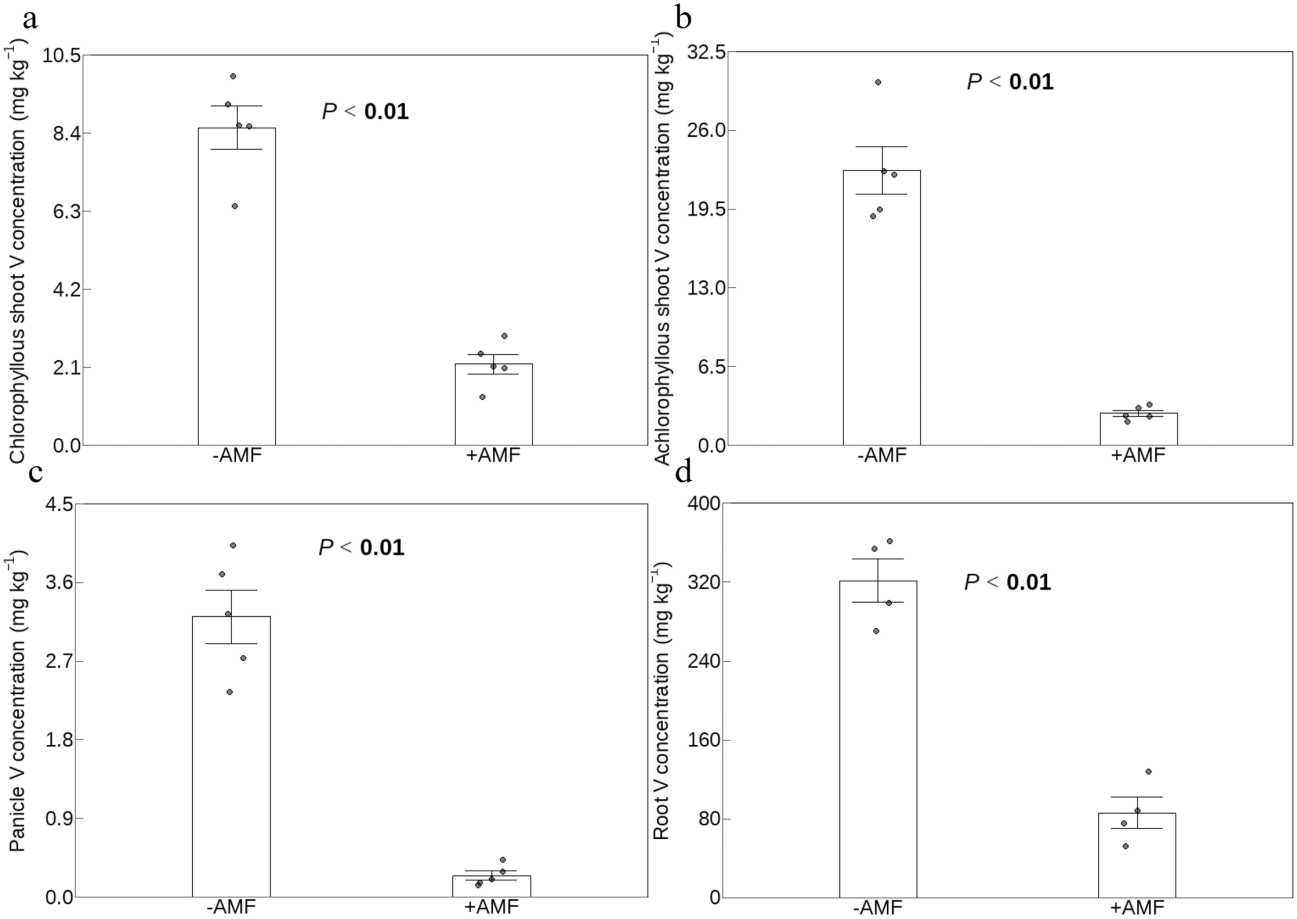
The effect of AMF on V concentrations of chlorophyllous shoots **(a)**, achlorophyllous shoots **(b)**, panicles **(c)** and roots **(d)**. The shoot of plants was divided into three sections: chlorophyllous shoots, achlorophyllous shoots and panicles; -AMF and +AMF: non-inoculated and inoculated with AMF, respectively; the error bars were calculated as the standard deviations.

V concentrations were significantly lower in +AMF plants under V stress than in -AMF plants, irrespective of shoot or root (**Figure 1**). The V concentration for +AMF treatment was as follows, going from highest to lowest: roots (85 mg kg^-1^) > achlorophyllous shoots (2.7 mg kg^-1^) > chlorophyllous shoots (2.2 mg kg^-1^) > panicles (0.3 mg kg^-1^). A similar trend in the treatment of -AMF was noted.

### 3.3 Plant growth and V content

**Figure 2:**
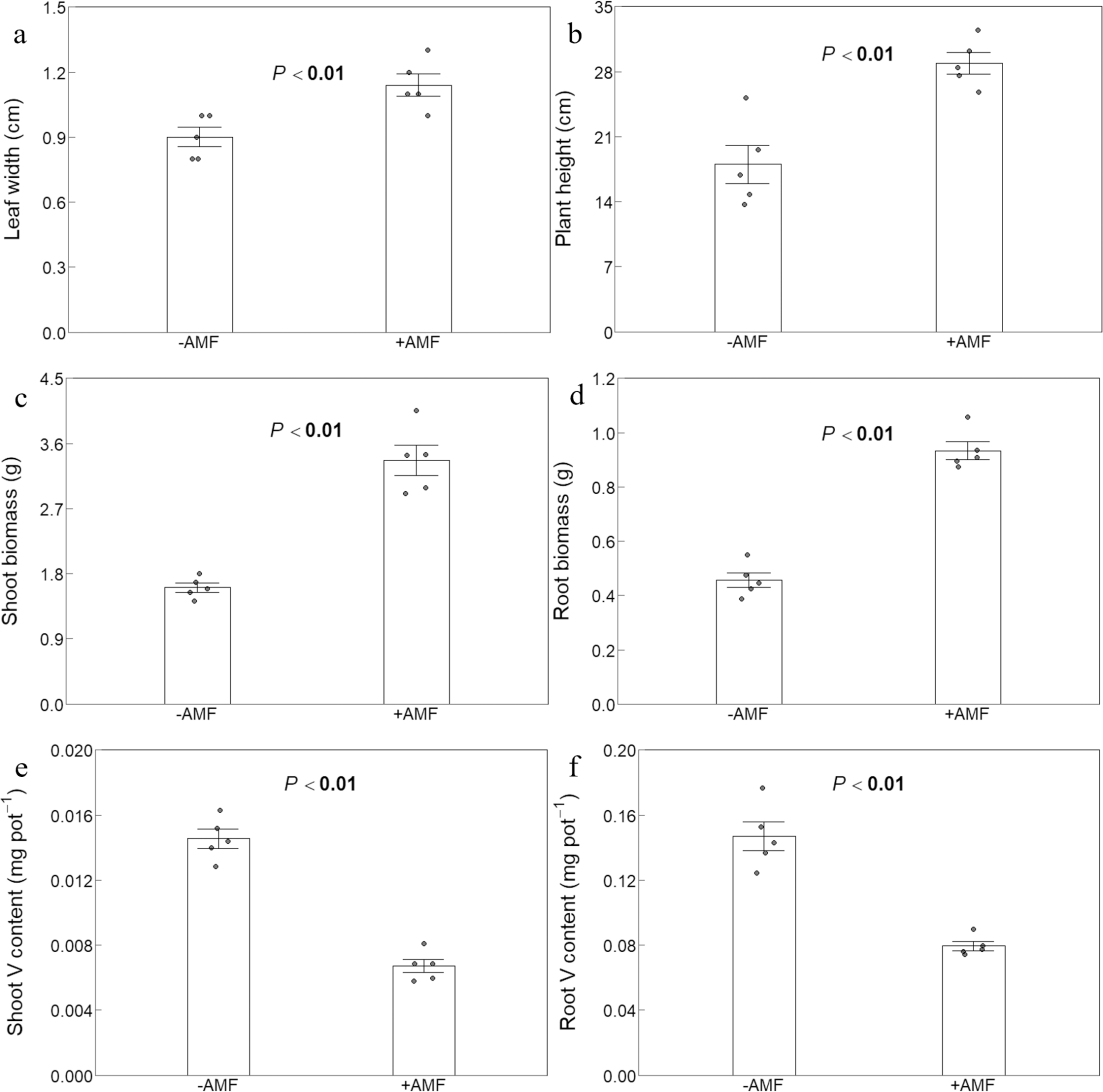
The effect of AMF on leaf width **(a)**, plant height **(b)**, shoot biomass **(c)**, root biomass **(d)**, shoot V content **(e)** and root V content **(f)**. -AMF and +AMF: non-inoculated and inoculated with AMF, respectively; the error bars were calculated as the standard deviations.

AMF inoculation resulted in wider leaves and taller plants under V stress conditions (**Figures 2a, b**). The shoot biomass of the +AMF treatment (3.4 g) was substantially greater than that of the -AMF treatment (2.1 g) in terms of biomass. The observed pattern was comparable to that of the root biomass. This showed that AMF inoculation can successfully increase the accumulation of both shoot and root biomass in plants under V stress (**Figures 2c, d**). The V content in both roots and shoots of +AMF plants was significantly lower compared to -AMF plants. It was noteworthy that regardless of whether AMF was present, the V content in the roots was approximately 10 times higher than in the shoots (**Figures 2e, f**).

### 3.4 Biological enrichment and translocation of V

**Figure 3:**
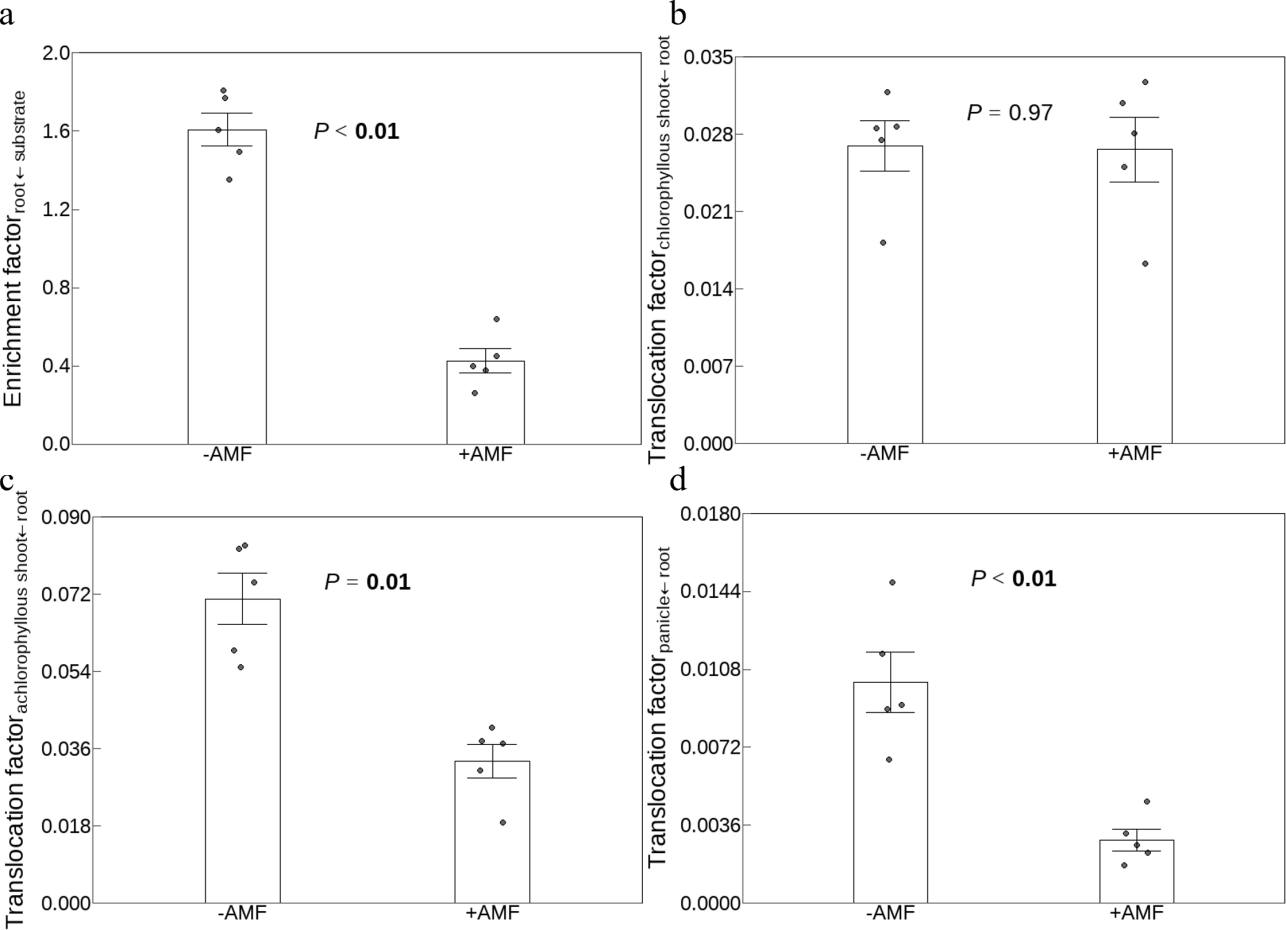
The effect of AMF on the enrichment factor **(a)**, translocation factor of chlorophyllous shoots **(b)**, achlorophyllous shoots **(c)** and panicles **(d)**. The shoot of plants was divided into three sections: chlorophyllous parts, achlorophyllous parts and panicles. -AMF and +AMF: non-inoculated and inoculated with AMF, respectively; the error bars were calculated as the standard deviations.

The enrichment factor was 1.6 for the -AMF treatment and 0.4 for the +AMF plant (**Figure 3a**). Similarly, +AMF plants had lower translocation factor for achlorophyllous shoots than -AMF plants (**Figure 3c**) but comparable translocation factor for chlorophyllous shoots with -AMF plants (**Figure 3b**). Furthermore, compared to -AMF plants, the translocation factor, which was determined by the V concentrations of panicles and roots, was lower in +AMF plants.

### 3.5 Subcellular distribution of V

**Figure 4:**
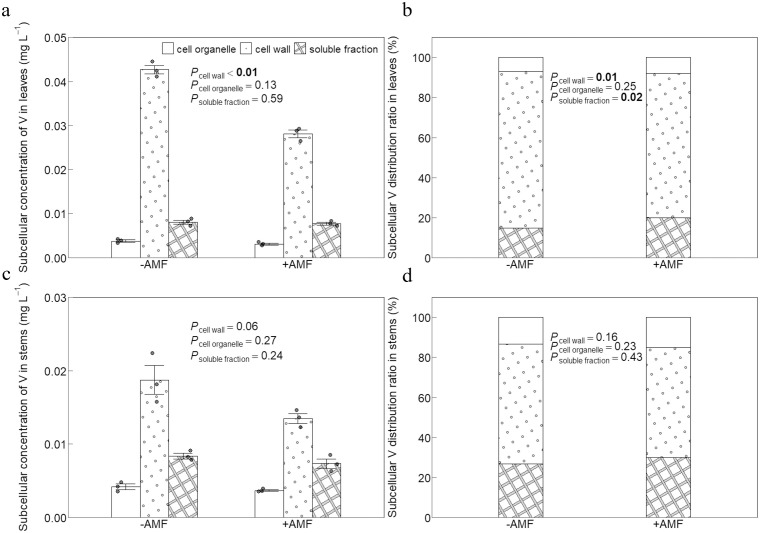
The effect of AMF on subcellular concentrations **(a)** and ratios **(b)** of V in leaves, and on subcellular concentrations **(c)** and ratios **(d)** of V in stems. -AMF and +AMF: non-inoculated and inoculated with AMF, respectively; *P*_cell wall_, *P*_cell organelle_ and *P*_soluble fraction_: significance of the difference between the -AMF and +AMF treatments in cell wall, cell organelle and soluble fraction.

Organelle and soluble fraction V concentrations did not significantly differ between +AMF and -AMF plants, but the V concentrations of cell wall in +AMF treatment were significantly lower than those in -AMF control (**Figure 4a**). The highest V ratio was observed in the cell wall of both +AMF and -AMF plants: 72% for +AMF and 78% for -AMF plants. The lowest V ratio was found in the cell organelles: 8% for +AMF and 7% for -AMF plants (**Figure 4b**).

The V concentrations in the stems and leaves showed a similar pattern, and the difference between the +AMF and -AMF plants was not statistically significant in the stems (**Figure 4c**). The V ratio for plants with and without AMF was as follows: cell wall > soluble fraction > cell organelles (**Figure 4d**).

### 3.6 Concentrations of plant thiol derivatives

**Figure 5:**
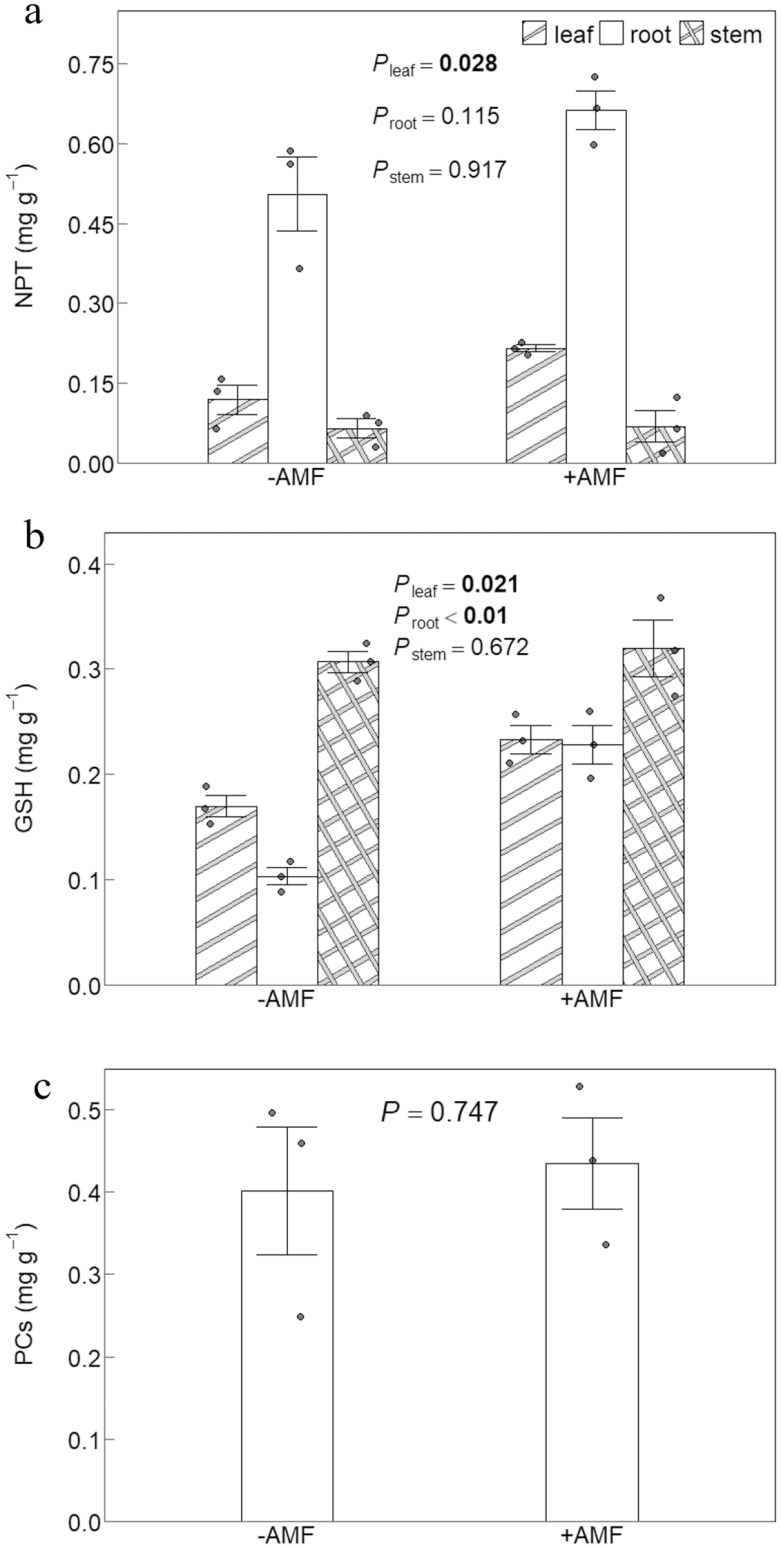
The effect of AMF on concentration of NPT **(a)**, GSH **(b)** and PCs **(c)**. -AMF and +AMF: non-inoculated and inoculated with AMF, respectively; *P*_leaf_, *P*_root_ and *P*_stem_: significance of the difference between the -AMF and +AMF treatments in leaf, root and stem; NPT: non-protein thiols; GSH: glutathione; PCs: phytochelatins; the error bars were calculated as the standard deviations.

The concentrations of non-protein thiols (NPT) in the leaves of +AMF plants were higher than those of -AMF plants (**Figure 5a**), as well as higher levels of glutathione (GSH) in the roots and leaves (**Figure 5b**). The two sets of plants had comparable levels of phytochelatins (PCs), despite the fact that PCs were not found in the shoots (**Figure 5c**). Under V stress, the roots had greater NPT concentrations than the shoots (**Figure 5a**), whereas the GSH levels in stems were greater than in leaves and roots (**Figure 5b**).

## 4 Discussion

Smelting, mining, and burning fossil fuels are examples of intensive industrial processes that can seriously pollute soil with V (Cao et al., 2017). It is essential to investigate whether and how AMF improve plant growth and V accumulation for creating remediation technologies for soils polluted with V (Wang X. W. et al., 2020). Our data revealed that AMF increased plant growth while paradoxically decreased V concentration in a V-hyperaccumulator candidate (green foxtail). The decoupling mechanism might stem from the fact that AMF reprogrammed V dynamics including V enrichment, translocation, distribution and chelation in plants (Dhalaria et al., 2020; Riaz et al., 2021).

### 4.1 AMF decoupled plant growth and V accumulation

In our previous study, we found that in constructed wetlands treating V-polluted water, AMF increased plant biomass, V concentrations and then V accumulation of reeds (Zhang et al., 2024). Our data of this study showed that AMF considerably increased growth indicators of green foxtail (**Figures 2a–d**). Unexpectedly, AMF decreased V concentrations of plant panicles, shoots and roots (**Figure 1**). This different effect of other AMF strains was also observed on growth and V concentrations of several crops. For example, *Funneliformis mosseae* BGCXJ01 had different effects on plant growth and V concentrations of alfalfa under V stress (1705 mg kg^-1^) (Qiu et al., 2021) and the strain of *Funneliformis mosseae* (BGC HUN03B) had such different effects on maize (V stress: 100, 200 and 400 mg kg^-1^) (Qiu et al., 2024). In addition, *Rhizophagus irregularis* had such different effects on plant growth and V concentrations in rye and sorghum (V stress: 350 mg kg^-1^) (Selim et al., 2021). Importantly, this led to a decrease in V accumulation indicated by V content (the product of biomass multiplied by V concentration) (**Figures 2e, f**). This means that AMF decoupled the growth and V accumulation of a V-hyperaccumulator candidate. This was in line with sorghum and maize, rather than other tested plants (Qiu et al., 2021; Selim et al., 2021; Qiu et al., 2024).

Under the stress of heavy metals, whether AMF decouple plant growth and accumulation of heavy metals might depend on several factors, including differences in plant species, AMF species, heavy metals and their intensity (Yang et al., 2015; Selim et al., 2021). For example, under arsenic stress, colonization of *Rhizoglomus irregulare* decouples biomass production from tissue arsenic concentration in roots and leaves of *Medicago sativa*, consistently enhancing growth while reducing arsenic accumulation (Li et al., 2021). Under chromium stress, AMF colonization elicits partitioned effects: it decouples biomass from metal concentration in shoots while concurrently promoting both parameters in roots (Kullu et al., 2020). Conversely, lead exposure reverses this pattern—AMF synchronously enhances shoot biomass and lead accumulation but decouples these responses in roots (Zhang et al., 2021). For cadmium, however, AMF consistently decouples biomass-metal relationships asynchronously across both roots and shoots, reducing tissue cadmium concentrations irrespective of growth stimulation (Zhang et al., 2019), This change may be attributed to AMF-induced alterations in soil properties (Zhao et al., 2024).While our study delineated AMF’s role under V stress, real-world applications face greater complexity: the ubiquitous co-occurrence of V with arsenic, chromium, cadmium, and lead establishes complex multi-metal matrices (Jiang et al., 2018). Deploying AMF for ecological restoration in such co-polluted systems demands holistic consideration of their ecological functionality—specifically, whether AMF decouple growth-accumulation of heavy metals under concurrent yet divergent heavy metal stressors. This necessitates future work elucidating: AMF’s priority regulation hierarchy in multi-metal systems and metal-metal interactions at AMF-soil interfaces.

### 4.2 AMF decreased V enrichment and root-to-shoot V translocation

The majority of heavy metals are not necessary for the physiological processes of plants (Li et al., 2015), leading to an enrichment factor lower than 1 (V concentration: plant root ≤ the environment). In our study, the enrichment factor of -AMF plants (1.6) was higher than 1 (**Figure 3a**). This was consistent with an investigation reporting that green foxtail was a V hyperaccumulator candidate (Aihemaiti et al., 2017). The enrichment of V likely results from plants absorbing vanadate via phosphate transporters, given the structural similarity between phosphate and vanadate (Zhang et al., 2021). Unlike -AMF plants, +AMF plants had an enrichment factor (0.4) lower than 1 (**Figure 3a**). This was consistent with the study conducted on *Medicago sativa* (Qiu et al., 2021), sorghum and rye (Selim et al., 2021). This negative effect of AMF was also observed on the enrichment factor of cadmium in maize (Kuang et al., 2025) and rice (Wang et al., 2014). The lower enrichment factor of +AMF plants might be attributed to that AMF exhibit high affinity for phosphate and enhance phosphate absorption, thereby competitively inhibiting V uptake through these shared transporters (Zhang et al., 2025a). In addition, the extraradical hyphae of AMF produced polyphosphates (Zu et al., 2015; Zhang et al., 2018), which may bind V within the hyphae and decrease their bioavailability and transport into the plant (Turnau et al., 1993). AMF might also secrete organic acids (e.g., citrate) and these exudates might chelate V in the soil (Cheng et al., 2025), reducing its bioavailability and uptake and then inhibiting V enrichment.

Comparing to -AMF plants, +AMF plant had a lower translocation factor for achlorophyllous shoots (**Figure 3c**). For chlorophyllous shoots, the translocation factor was comparable between the two sets of plants (**Figure 3b**). This indicated that AMF encouraged the release of V from achlorophyllous shoots instead of chlorophyllous ones. More data is required to substantiate this theory, taking into account various plant and AMF strain species. Furthermore, +AMF plants displayed lower values than -AMF plants when the translocation factor was calculated using the V concentrations of panicles rather than leaves and stems (**Figure 3d**). These results showed that the translocation of V from the roots to the green foxtail’s productive organs was decreased by AMF. This was in agreement with a recent study reporting that AMF lowered the translocation factor of V in maize (Qiu et al., 2024). Importantly, this negative effect of AMF was also observed on the translocation of other heavy metals (Hu et al., 2022), such as arsenic in white clover and ryegrass (Dong et al., 2008) and cadmium in rice (Wang et al., 2014). A recent study found that the combined application of AMF and zinc oxide nanoparticles in cadmium-polluted soil can more effectively reduce the translocation of cadmium to wheat grains and ensure compliance with China’s food safety threshold (< 0.2 mg kg^-1^) (Yang et al., 2025). Scale lab findings to real-world soils with variable metal co-contamination is needed in future studies.

### 4.3 AMF retained less V in the cell wall of leaf cells

The main components of plant cell walls are proteins and polysaccharides, such as cellulose, hemicellulose, and pectin, which offer a large number of heavy metal binding sites and efficiently restrict the transmembrane movement of heavy metal ions (Bashline et al., 2014). This restriction shields subcellular structures from the harmful effects of heavy metals (Hou et al., 2020a). According to our research, the concentration and ratio of V in leaves and stems under V stress in both +AMF and -AMF plants followed the following pattern: cell wall > soluble fraction > organelles (**Figure 4**). This was consistent with earlier research reporting that the cell wall of maize leaves under V stress had a significantly higher V concentration than other subcellular components (Hou et al., 2020b), indicating that the cell wall serves as the first line of defense against V stress (Hou et al., 2020a). Retaining more V in the cell wall helps to reduce the damage of V to plant cells and enhances the plant’s tolerance to V (Riaz et al., 2021), consistent with recent studies on other metals (Chen et al., 2025; Yu et al., 2025). The subcellular V concentrations in the cell wall of +AMF plant leaves were lower than those of -AMF plants (**Figures 4a,b**). We did not find any other studies investigating the effects of AMF on the subcellular distribution of V. In spite of this, AMF were observed to have a similar role on the subcellular distribution of cadmium in kenaf (Pan et al., 2023) and rice (Li et al., 2016). This role of AMF may arise from their induction of plant cell-wall biosynthetic genes—such as cellulose synthases genes (Mendoza-Soto et al., 2022)—which increases the abundance of pectin, cellulose and hemicellulose (Huang et al., 2018), thereby diluting V content and lowering V concentrations in the leaf cell wall.

### 4.4 AMF upregulated NPT and GSH of leaves and roots

V has been shown to promote the accumulation of thiol derivatives in plants, including PCs and GSH (Hou et al., 2020a). These compounds directly complex with free V ions, thereby reducing their biological toxicity. The resulting V–thiol complexes are then sequestered in vacuoles (Khoudi, 2021), effectively compartmentalizing V (Shireen et al., 2021). We discovered that +AMF plant leaves exhibited higher concentrations of NPT and GSH in their leaves than -AMF plants (**Figures 5a, b**). These results were comparable to that of pigeonpea under cadmium stress, where AMF inoculation resulted in a noticeably greater accumulation of NPT compared to plants that were not inoculated (Bisht and Garg, 2022; Paladines-Beltrán et al., 2025). Other research has also demonstrated that AMF inoculation has a positive impact on plants’ accumulation of non-protein thiols under arsenic and cadmium stress (Sharma et al., 2017; Bisht et al., 2023). AMF might promote concentrations of these thiol derivatives by upregulating of the expression of genes encoding glutathione synthetase (Li H. et al., 2022), plant cheloprotein synthase, and transport proteins, thereby facilitating the synthesis of GSH and PCs.

## 5 Conclusion

Our study demonstrated that AMF decoupled the growth and V accumulation of a V-hyperaccumulator candidate (green foxtail), providing novel insights into phytoremediation of V-polluted soils. AMF played this role by reprogramming V dynamics in plants. They decreased V enrichment in roots and translocation to achlorophyllous shoots (rather than chlorophyllous shoots). The green foxtail plants colonized by AMF retained less V in leaf cell walls, compared to their non-colonized counterparts. AMF upregulated the accumulation of thiol derivatives in plant leaves, such as NPT and GSH (rather than PCs). These findings redefine AMF’s role in phytoremediating soils polluted with V, emphasizing the effect of AMF on V dynamics in plants. Our study did not explore the role of AMF in soils with different levels of V pollution, nor did it investigate AMF’s priority regulation hierarchy in multi-metal systems, and metal-metal interactions at AMF-soil interfaces. In addition, our laboratory data needs verification in field conditions. Based on these insights, future research should focus on: 1) validating the dose-dependent effects of AMF across a broader gradient of soil V contamination levels; 2) elucidating AMF’s priority regulation hierarchy and the underlying molecular mechanisms in multi-metal co-contaminated systems; and 3) translating these laboratory findings into field-scale applications for sustainable phytoremediation.

## Data Availability

The datasets presented in this study can be found in online repositories. The names of the repository/repositories and accession number(s) can be found in the article/supplementary material. The data that support the findings of this study are openly available in Zenodo at https://doi.org/10.5281/zenodo.16631239.
